# Screening and Identification of Survival-Associated Splicing Factors in Lung Squamous Cell Carcinoma

**DOI:** 10.3389/fgene.2021.803606

**Published:** 2022-01-20

**Authors:** Min Chen, Rui Zhu, Fangzhou Zhang, Liucun Zhu

**Affiliations:** ^1^ School of Life Sciences, Shanghai University, Shanghai, China; ^2^ School of Materials Science and Engineering, Institute of Materials, Shanghai University, Shanghai, China; ^3^ Shaoxing Institute of Technology, Shanghai University, Shanghai, China

**Keywords:** lung squamous cell carcinoma, alternative splicing, splicing factor, bivariate cox regression, bipartite graph

## Abstract

Lung squamous cell carcinoma (LUSC) is a disease with high morbidity and mortality. Many studies have shown that aberrant alternative splicing (AS) can lead to tumorigenesis, and splicing factors (SFs) serve as an important function during AS. In this research, we propose an analysis method based on synergy to screen key factors that regulate the initiation and progression of LUSC. We first screened alternative splicing events (ASEs) associated with survival in LUSC patients by bivariate Cox regression analysis. Then an association network consisting of OS-ASEs, SFs, and their targeting relationship was constructed to identify key SFs. Finally, 10 key SFs were selected in terms of degree centrality. The validation on TCGA and cross-platform GEO datasets showed that some SFs were significantly differentially expressed in cancer and paracancer tissues, and some of them were associated with prognosis, indicating that our method is valid and accurate. It is expected that our method would be applied to a wide range of research fields and provide new insights in the future.

## Introduction

Lung cancer is one of the most common malignant tumors, and about 85% of cases are non-small cell lung cancer (NSCLC) (Wang et al., 2019). According to pathological classification, NSCLC can be divided into lung squamous cell carcinoma (LUSC) and lung adenocarcinoma (LUAD) (Cheng et al., 2019). Compared with LUAD, patients with LUSC have a poorer treatment outcome and prognosis (Li et al., 2018). In recent years, targeted therapies for specific genes have greatly improved the living conditions of patients with advanced LUAD. However, LUSC patients respond poorly to targeted therapies due to the lack of driver mutations, and the specific molecular mechanisms of LUSC pathogenesis and progression have not been systematically assessed. As a result, further exploration of the molecular mechanisms underlying the development of LUSC is essential for the development of more effective therapeutic regimens.

Alternative splicing (AS) is an important post-transcriptional regulatory mechanism. A single gene can generate more than one mRNA transcript through AS, and each mRNA transcript encodes a protein with a different structure and function (Baralle and Giudice, 2017). More than 95% of human genes experience AS under normal physiological conditions. On the one hand, the AS process regulates the tissue-specific and stage-specific expressions of specific genes during human development (Xu et al., 2002; Pan et al., 2008) and is essential for normal biological processes, such as hematopoiesis (Wong et al., 2018), brain development (Matsuda et al., 2019), and muscle function (Nakka et al., 2018). On the other hand, abnormal AS triggers a series of tumor-related processes, including cell proliferation (Xie et al., 2019), apoptosis (Tyson-Capper and Gautrey, 2018), epithelial-mesenchymal transition (EMT) (Pradella et al., 2017), and tumor invasion and metastasis (Chen et al., 2017; Wang et al., 2017) in response to hypoxia (Han et al., 2017), thereby promoting malignant cell transformation and providing a survival advantage (Climente-González et al., 2017; Moncada et al., 2020). The AS process is regulated by splicing factors (SFs), and abnormal expression of SFs is the main contributor to overall changes in alternative splicing events (ASEs) in malignancies (David and Manley, 2010; Dvinge et al., 2016; Su et al., 2018). Therefore, exploring abnormal ASEs and SFs in malignant tumors may provide new insights into the mechanisms of tumorigenesis and progression.

Recent studies have paid more attention to assessing the clinical significance of ASEs and SFs in cancers and their potential pathogenic pathways and regulatory networks. The abnormal ASEs and SFs, which make network dysregulated, have been shown to modulate malignant transformation of cells and epithelial-mesenchymal transition (Sveen et al., 2016). Several excellent studies have also discussed the role of SFs in DNA damage (Shkreta and Chabot, 2015) or in carcinogenesis and anticancer therapies (Miura et al., 2012; Shkreta et al., 2013). However, SFs have the potential to become molecular markers and therapeutic targets for malignancies (Anczukow and Krainer, 2016; Yuan et al., 2017; Park et al., 2019). Although there is an increasing systematic analysis of AS signatures and the effect of SFs in colorectal cancer, glioblastoma, breast cancer, and ovarian cancer (Dorman et al., 2014; Suo et al., 2015; Zong et al., 2018), the analytical methods for identifying tumor-associated SFs remain deficient. Only univariate difference and survival analysis were performed in these studies (Zhu et al., 2018; Hu et al., 2019; Zhao et al., 2020). However, biological processes are complex and are mostly regulated by multiple factors rather than a single factor. It is indicated that, as a whole, some factors would have a high correlation with the tumor process, but this would show a low correlation when they are separated. Hence, we propose an analysis method based on synergy to screen key factors that regulate the initiation and progression of LUSC. We first screened the ASEs associated with overall survival (OS-ASEs) from combinations consisting of two ASEs using bivariate Cox regression and AUROC. Then an association network consisting of OS-ASEs, SFs, and their targeting relationship was constructed to identify key SFs. This method can screen a relatively complete set of OS-ASEs to a certain extent, thereby improving the completeness for subsequent screening of key SFs and providing new ideas for LUSC mechanism research.

## Materials and Methods

### Data Collection and Preprocessing

Clinical information and expression levels of LUSC patients (generated by RNA-seq) were collected from The Cancer Genome Atlas (TCGA) database. Additionally, ASEs data were retrieved from the TCGASpliceSeq database (Ryan et al., 2016). In TCGASpliceSeq, the Percent Spliced In (PSI) values are computed for each possible splice event in each gene. PSI is the ratio of reads indicating the presence of a transcript element versus the total reads covering the event. The cross-platform validation set, including GSE157010, GSE3268, and GSE6044 (Supplementary Table S1), was downloaded from the NCBI-GEO database (Barrett et al., 2013). SFs are protein factors involved in the splicing process of pre-RNA. A total of 404 SFs were collected in this study (Wu et al., 2020), as shown in Supplementary Table S2.

**FIGURE 1:**
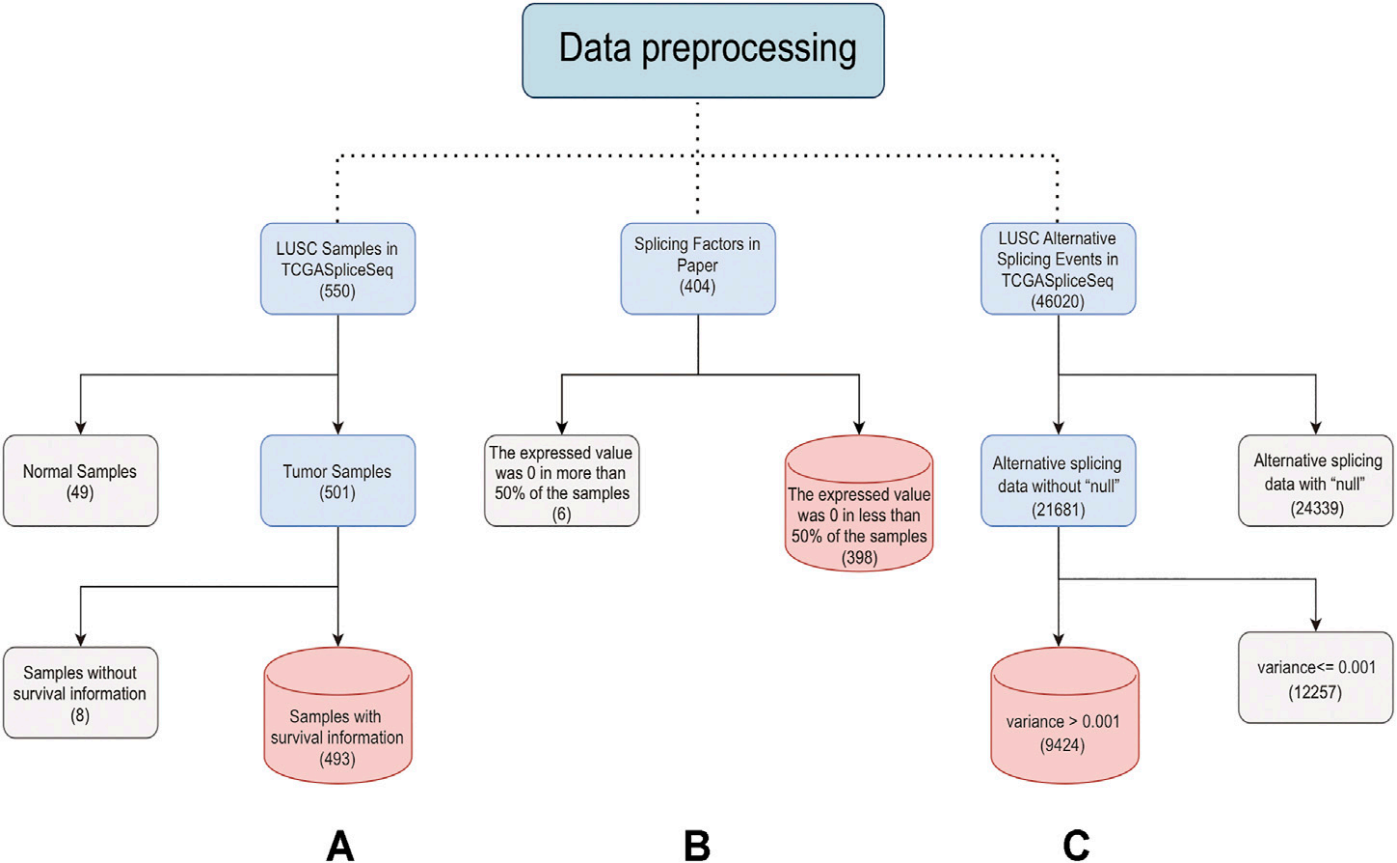
Steps of data preprocessing. The solid line represents the preprocessing process. The light blue box represents the data to be processed. The gray box represents the rejected data. The red box represents the last retained data. The numbers in brackets represent the data amount. **(A)** is the preprocessing process of samples, **(B)** is the preprocessing process of SFs, and **(C)** is the preprocessing process of ASEs.

The TCGA database included 550 LUSC samples, 501 of which were tumor samples. After removing 8 samples with no clinical information, 493 tumor samples were retained for subsequent analysis (Supplementary Table S3). The TCGASpliceSeq database contained a total of 46,020 ASEs for LUSC, of which 9424 ASEs were retained for subsequent analysis by removing ASE containing “null” and then excluding ASEs with variances less than 0.001 in all samples (Supplementary Source Code S1) (Supplementary Source Code S2). The distinguishable visualization UpSet plot, generated by *UpSetR* (version 1.4.0) (Wang et al., 2021), was used to quantitatively analyze the intersections among the seven types of ASEs in LUSC. The expressions of 404 SFs were extracted after being normalized by log2 (FPKM+1) (Bullard et al., 2010). SFs with expression values of 0 in half of the samples were excluded, and 398 SFs were finally retained for subsequent analysis (Figure 1). The GSE157010 dataset constitutes 235 LUSC tumor samples, each containing clinical information. The GSE3268 dataset represents 5 tumor samples from LUSC patients and paired normal samples. The GSE6044 dataset includes 5 normal samples and 15 tumor samples. Ten of these 15 tumor patients have not received platinum-based therapy, and the other five have. Probe IDs for each GEO dataset were converted to Ensembl ID. When multiple probes correspond to an Ensembl ID, only the probe with the highest mean is retained. The batch correction was performed to eliminate the batch effect of three datasets using normalizeBetweenArrays function of *limma* (version 3.46.0).

### Methods for Screening Alternative Splicing Events Associated With Overall Survival

In order to investigate the prognostic value of ASEs in LUSC patients, all bivariate ASEs combinations were first constructed. Then Cox proportional risk hypothesis tests and bivariate Cox proportional risk regressions were performed using the survival package in R (Bradburn et al., 2003). The significance of the independent variables in the regressions was tested using likelihood ratio tests (Hazra and Gogtay, 2017). Additionally, the area under the receiver operating characteristic curve (AUROC) was used to show the sensitivity and specificity of the bivariate combination model in predicting OS (Linden, 2006). Values greater than 0.8 were considered excellent combinations. The two indicators mentioned above, the *p*-value of the likelihood ratio test and the AUROC, were used to screen OS-ASEs.

### Methods for Association Network Construction and Analysis

Spearman correlation analysis was performed to explore the correlation between the PSI values of OS-ASEs and the expression levels of SF genes (Bishara and Hittner, 2012). The correlation networks visualization was visualized by *EVeen* (Chen T. et al., 2021) with SFs and the OS-ASEs as vertices and the Spearman significant correlation between them as edges. It is assumed that the value of a vertex in a network depends first on its position in the network. More central vertex indicates a greater impact on the structure and function of the network (Kitsak et al., 2010). The importance of a vertex in the network is usually expressed by degree centrality, which is the number of connected edges of the vertex in the network (Freeman, 1978).

### Validation Methods for Alternative Splicing Events and Splicing Factors Functions

In order to identify potential mechanisms of OS-ASEs in LUSC, the survival-related genes were analyzed by Gene Ontology (GO) enrichment analysis and Kyoto Encyclopedia of Genes and Genomes (KEGG) enrichment analysis, which were both done by *DAVID* (Huang et al., 2009). The results of KEGG analysis were presented by bubble plots generated by *ggplot2* (version 3.3.5). The results of GO analysis were visualized by a web tool *Revigo*, which shows the cluster representatives in a two-dimensional space derived by applying multidimensional scaling to a matrix of the GO terms’ semantic similarities (Supek et al., 2011).

In order to validate the function of SFs, violin plots visualized by *ggplot2* (version 3.3.5) were used for verifying the difference in the expression of SFs in tumor and normal tissues. The paired-samples *t* test was used to test the significance of the difference.

The Kaplan-Meier (KM), generated by *survival* (version 3.2-11) and *survminer* (version 0.4.9), was applied to validate the prognostic effect of SFs (Supplementary Source Code S3) (Dinse and Lagakos, 1982). The log-rank test was used to test the significance of differences in survival between high- and low-risk patients (Mantel, 1966). The *p*-value < 0.05 was considered statistically significant in this study.

## Results

### Clinical Characteristics of the Lung Squamous Cell Carcinoma Cohort

The current study included a total of 493 LUSC patients from the TCGA database, and the characteristics and clinical information of these patients are listed in Table 1. There were 365 men and 128 women among these patients. With a median age of 68 (ranging from 39 to 85 years old), the mean survival time of patients was 1,044 days (ranging from 4 to 4,765 days). It is worth noting that the survival time of patients is censored data. The patient mortality rate of 43% confirms that LUSC is a tumor with a high mortality rate. The LUSC tumor staging data show that most patients are in stages I or II. Stage I tumors are usually small, without lymph nodes and distant metastases, and can be completely removed by surgery. In contrast, higher stages mean that the tumor is more progressive.

**TABLE 1:** Clinical characteristics of 493 LUSC patients in the TCGA database.

Characteristics	Groups	No. of patients	%
Sex	Male	365	74
	Female	128	26
Age at diagnosis	Median	68	
	Range	39–85	
	<61	107	22
	≥61	381	77
	Unknown	5	1
Vital status	Alive	268	54
	Dead	211	43
	Unknown	14	3
Stage	I	241	48.88
	II	158	32.05
	III	83	16.84
	IV	7	1.42
	Unknown	4	0.81
T category	T1	114	23
	T2	286	58
	T3	70	14
	T4	23	5
N category	N0	316	64
	N1	127	26
	N2	40	8
	N3	5	1
	NX	5	1
M category	M0	405	82
	M1	7	1
	MX	77	16
	Unknown	4	1

**TABLE 2:** Top 10 SFs for degree centrality.

Rank	Ensembl	Symbol
1	ENSG00000130332	LSM7
2	ENSG00000169976	SF3B5
3	ENSG00000163634	THOC7
4	ENSG00000051596	THOC3
5	ENSG00000108561	C1QBP
6	ENSG00000137168	PPIL1
7	ENSG00000079134	THOC1
8	ENSG00000139343	SNRPF
9	ENSG00000108883	EFTUD2
10	ENSG00000123154	WDR83

### Overview of Alternative Splicing Events in the Lung Squamous Cell Carcinoma Cohort

**FIGURE 2:**
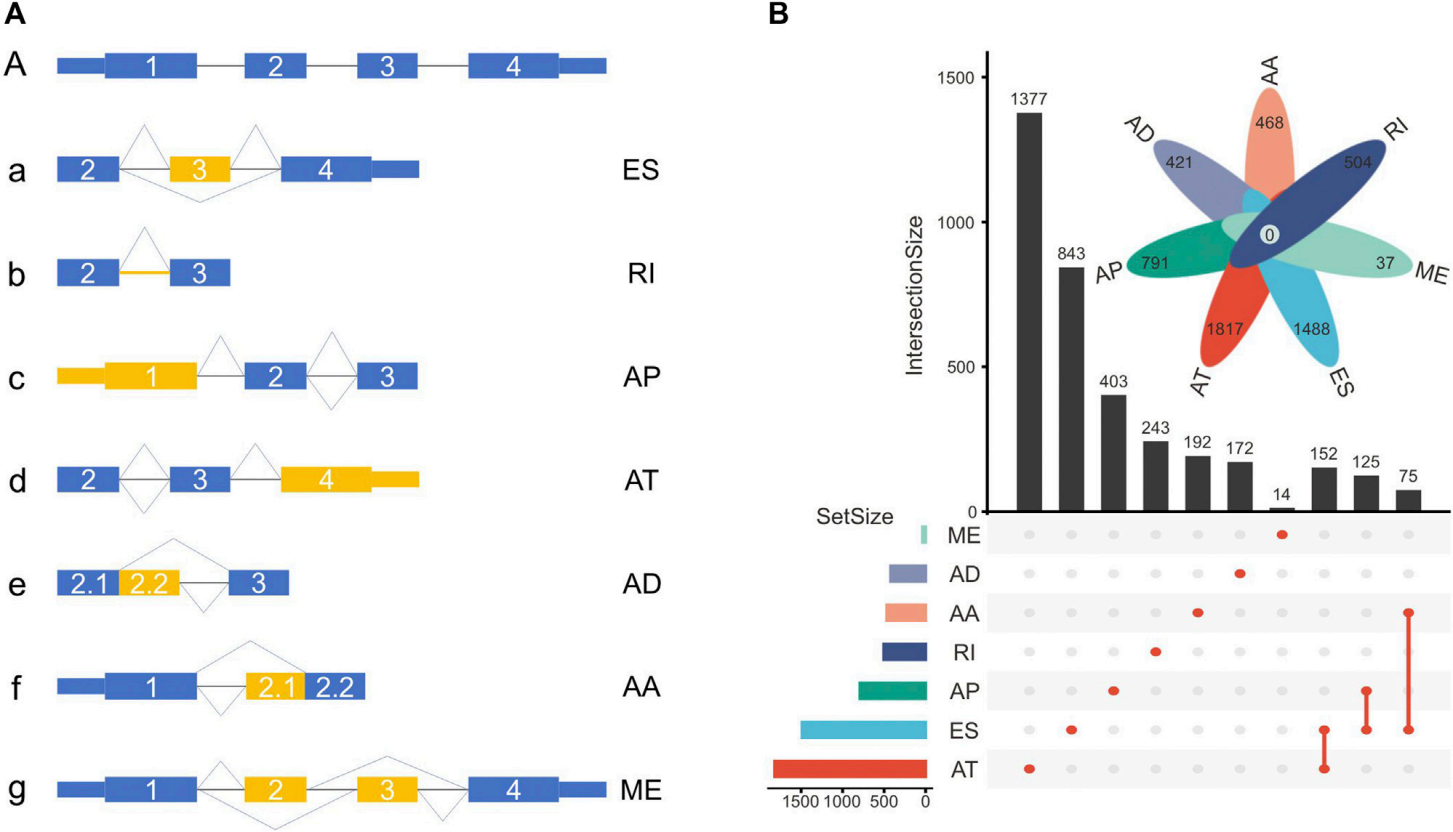
**(A)** Schematic representation of ASEs, including exon skipping (ES), intron retention (RI), alternative promoter (AP), alternative terminator (AT), alternative donor (AD) site, alternative acceptor (AA) site, and mutually exclusive (ME) exon. **(B)** The number of genes with ASEs in LUSC, with 3876 ATs in 1817 genes, 2048 ESs in 1488 genes, 1761 APs in 791 genes, 656 RIs in 504 genes, 531 AAs in 468 genes, 515 ADs in 421 genes, and 37 MEs in 37 genes.

The TCGASpliceSeq database recorded seven types of ASEs, including exon skipping (ES), mutually exclusive (ME) exons, intron retention (RI), alternative promoter (AP), alternative terminator (AT), alternative donor (AD) site, and alternative acceptor (AA) site (Figure 2A).

In this cohort, a total of 9424 ASEs were in 4246 genes, with 3876 ATs in 1817 genes, 2048 ESs in 1488 genes, 1761 APs in 791 genes, 656 RIs in 504 genes, 531 AAs in 468 genes, 515 ADs in 421 genes, and 37 MEs in 37 genes. Multiple ASEs can occur in a single gene (Figure 2B).

### Screening and Analysis of Alternative Splicing Events Related to Survival

Due to the complexity of biological processes, synergistic interactions between genes are more prevalent. To accurately screen OS-ASEs, we employed the *p*-value of the likelihood ratio test in bivariate Cox proportional risk regression and AUROC as screening criteria ( P<0.05,AUROC>0.8 ). Consequently, 1118 combinations of OS-ASEs were screened, including 953 non-redundant ASEs.

**FIGURE 3:**
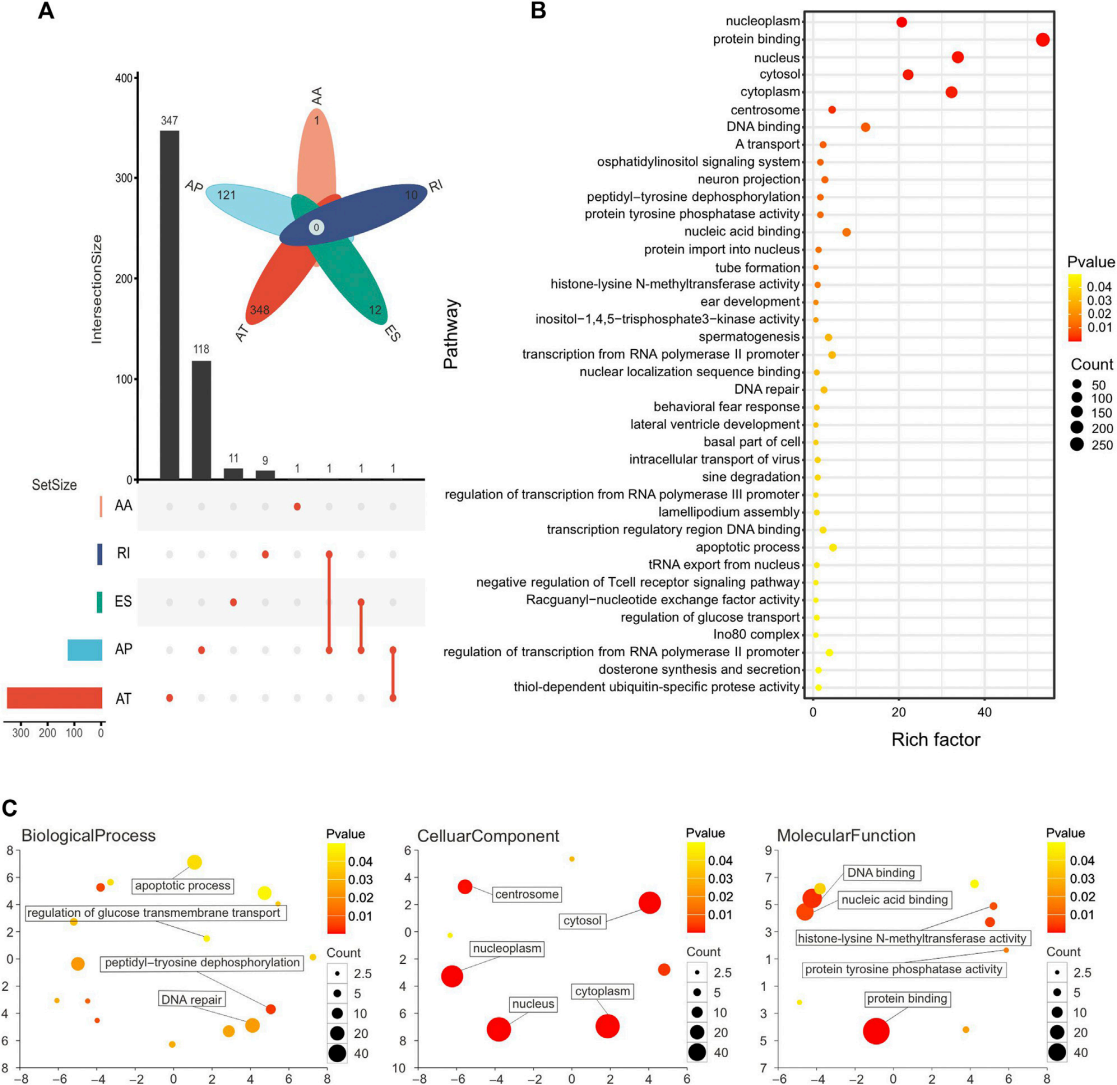
**(A)** Number of genes with OS-ASEs in LUSC, with 689 ATs in 348 genes, 241 APs in 121 genes, 12 ESs in 12 genes, 10 RIs in 10 genes, and 1 AA in 1 gene. **(B)** Pathway enrichment analysis of genes with OS-ASEs. Larger dots represent more genes enriched in the pathway and vice versa. A smaller *p*-value is represented when the color of the dot is closer to blue, and a larger *p*-value is represented when the color of the dot is closer to red. **(C)** Functional enrichment analysis of genes with OS-ASEs. The scatterplot shows the cluster representatives in a two-dimensional space derived by applying multidimensional scaling to a matrix of the GO terms’ semantic similarities. The dot represents all GO items, and its size is related to the number of genes enriched in that GO term. The color of dots is related to the *p*-value. A smaller *p*-value is represented when the color of the dot is closer to blue, and a larger *p*-value is represented when the color of the dot is closer to red.

A total of 953 OS-ASEs were detected in 489 genes. More specifically, there were 689 ATs in 348 genes, 241 APs in 121 genes, 12 ESs in 12 genes, 10 RIs in 10 genes, and 1 AA in 1 gene. Two splice types, AD and ME, were not included (Figure 3A). Next, in order to understand the function of the genes corresponding to OS-ASEs, KEGG analysis and GO analysis were performed. KEGG analysis demonstrated that these genes were enriched in histone-lysine N-methyltransferase activity, protein tyrosine phosphatase activity, and DNA repair and apoptosis pathways. These pathways are closely associated with cancer progression (Östman et al., 2006; Wong, 2011; Jeggo et al., 2016; Husmann and Gozani, 2019). A recent study has shown that histone-lysine N-methyltransferase is a key driver for the induction of LUSC (Figure 3B) (Yuan et al., 2021). GO analysis revealed that these genes were enriched in both the nucleus and cytoplasm and play a role in protein binding, nucleic acid binding, and histone lysine N-methyltransferase activity. These genes are involved in important biological processes such as DNA repair, peptidyltryosine dephosphorylation, and apoptosis (Figure 3C).

### Construction and Analysis of the Association Network Between Splicing Factors and Alternative Splicing Events

**FIGURE 4:**
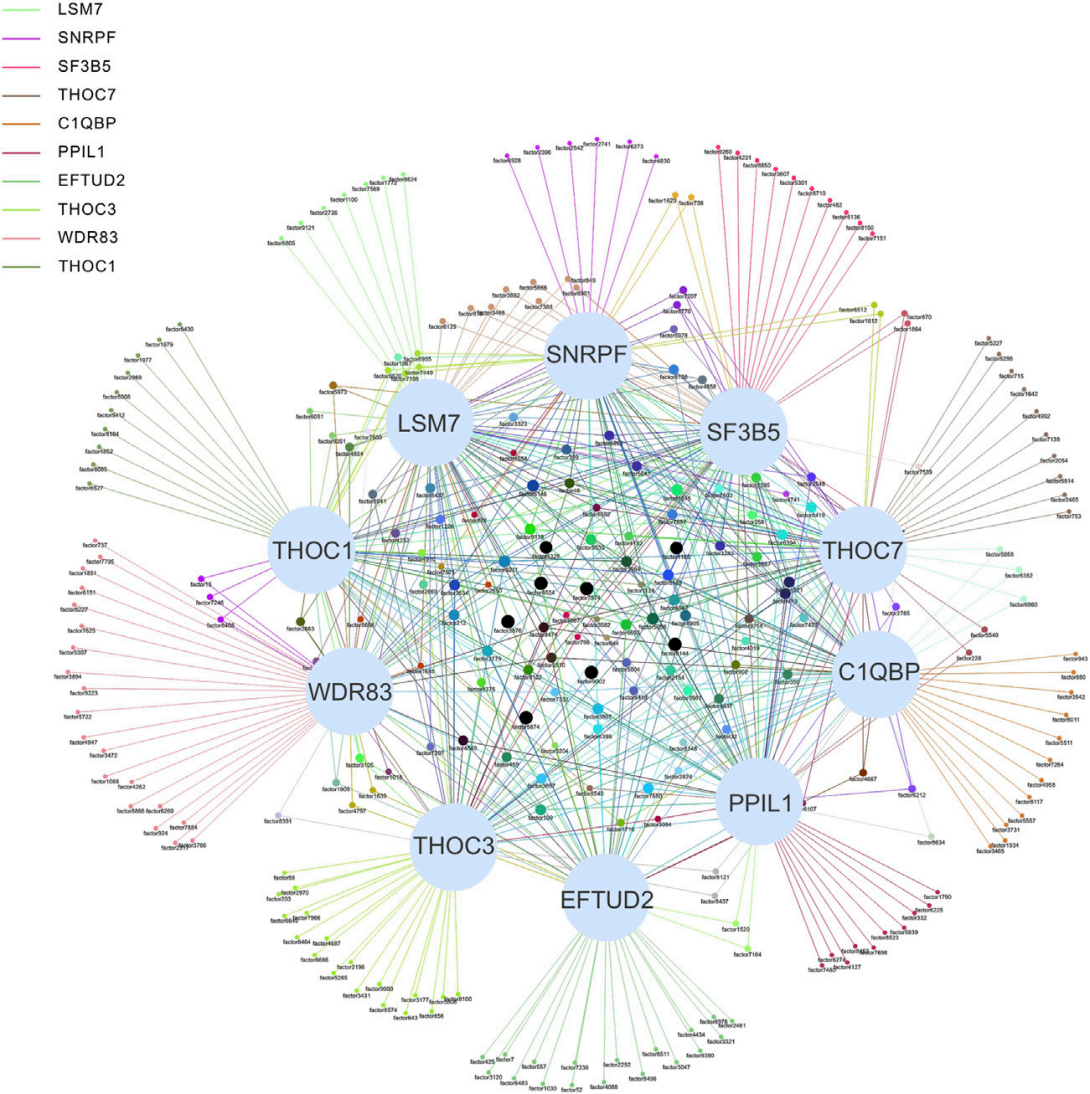
Network diagram of the top 10 SFs and OS-ASEs. The 10 large light blue dots represent 10 SFs, the small dots represent ASEs, and the edges represent significant correlations between the two dots. Edges of different colors represent associations with different SFs. When an ASE is associated with multiple SFs, the color of the edge is a superposition of the corresponding multiple colors. The correspondence between the factor and ASE is shown in Supplementary Table S3.

Systems biology is the study of the composition and interrelationships of the biological systems and is widely used in the study of gene networks (Hood, 2003). For our association network, identifying key vertices is an important way to find key SFs (Zhao and Liu, 2019). The association network was formed with 489 ASEs and 398 SFs as vertices and 9414 pairs of significant correlations as edges (Figure 4). The degree distribution is shown in Supplementary Figure S1. The average degree of the top 10 vertices in this network is 69, and the average degree of the remaining vertices is 22, indicating that the top 10 SFs are associated with more ASEs and is important in this network. Therefore, we consider these 10 SFs as key SFs. (Table 2)

### Validation of Splicing Factors

**FIGURE 5:**
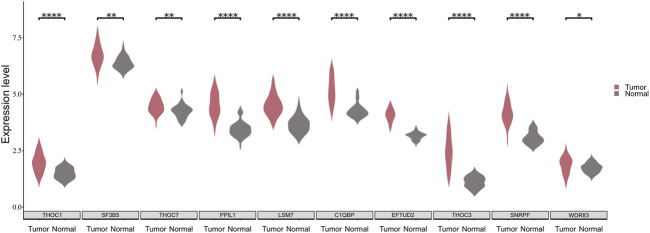
The expression distribution of 10 SFs between cancerous and paraneoplastic tissues in the TCGA dataset. The horizontal axis represents the expression of genes. The vertical axis shows the 10 key SFs.

To verify the validity of the above approach, we analyzed the expression patterns of the 10 SFs in the TCGA-LUSC dataset. It is noticed that a significant difference exists in the expression of the 10 SFs between cancerous and paracancerous tissues (Figure 5). Moreover, patients were divided into two groups according to the expression of SFs, and the difference of survival time between them was analyzed with KM curves. It is found that 5 of these 10 SFs are significantly associated with the prognosis of LUSC patients. (Supplementary Figure S2).

In order to further assess the applicability of our approach, three cross-platform datasets from the GEO dataset were recruited. The GSE157010 dataset matches 9 SFs, 6 of which exhibit prognostic function (Supplementary Figure S3). In the GSE3268 and GSE6044 datasets, the matched SFs are differentially expressed in normal and tumor samples (Supplementary Figure S4). In the GSE6044 dataset, the expression levels of SFs patients who received platinum-based therapy are slightly decreased compared with that of patients who did not, which is closer to the expression level in normal tissues (Supplementary Figure S5).

## Discussion

In this research, bivariate Cox regression and the systems biology approach were employed to detect OS-ASEs and SFs associated with LUSC. The results showed that all 10 candidates (SFs) were expressed at significantly higher levels in tumor samples than in paracancerous tissues in both the TCGA-LUSC and GEO datasets. Moreover, 7 of these SFs were associated with the overall survival time in tumor patients in one or more datasets. These results are consistent with the currently known characteristics of tumor-associated genes (Givechian et al., 2018; Qi et al., 2018). It is found that 3 of the 10 SFs are reported to be connected with lung cancer, namely LSM7, C1QBP, and THOC1. Specifically, LSM7 is a prognosis-related key gene and mediates autophagy in LUSC, with significant expression differences between tumor and normal tissues (Gatica et al., 2019; Li et al., 2020); C1QBP is involved in various cellular processes, including mRNA splicing, ribosome biosynthesis, protein synthesis in mitochondria, apoptosis, transcriptional regulation, and viral infection, and its expression correlated with the prognosis of patients with lung, breast, and colon tumors (Saha et al., 2019); THOC1 is down-regulated in lung cancer cell lines SPC-A1 and NCI-H1975, and its overexpression inhibits cell proliferation, induces G2/M cell cycle arrest, and promotes cell apoptosis (Wan et al., 2014). THOC1 also inhibits the proliferation of tumor cells in hepatocellular carcinoma and prostate cancer (Liu et al., 2015; Cai et al., 2020). The above evidence suggests that our method is reliable and accurate.

In addition, we identified 7 new SFs, 6 of which, including SF3B5, THOC7, THOC3, SNRPF, EFTUD2, and WDR83, were reported to be associated with other tumors. It has been suggested that SF3B5 is a key prognostic factor in ovarian cancer (Ouyang et al., 2021). Studies have shown a relationship between the downregulation of THOC7 and the activation of tumorigenic pathways in cervical cancer (Lando et al., 2013; Lando et al., 2015). THOC3 is involved in the THO subcomplex and is necessary for coupled mRNA transcriptional extension and nuclear export, and its expression is significantly elevated in glioma cells (Chen Z. et al., 2021). SNRPF is aberrantly expressed in human glioma. *In vitro* experiments have revealed that ubiquitin carboxy-terminal hydrolase isozyme L5 could inhibit human glioma cell migration and invasion by downregulating SNRPF (Ge et al., 2017). EFTUD2 is markedly overexpressed in hepatocellular carcinoma tissues. High expression of EFTUD2 in hepatocellular carcinoma patients is associated with clinical features and is pivotal in hepatocellular carcinoma cell proliferation and cell cycle course (Lv et al., 2021). As the NAT of WDR83, the protein-coding gene, deoxyhypusine synthase, concordantly regulates the expressions of WDR83 mRNA and protein. Conversely, WDR83 also regulates deoxyhypusine synthase by antisense pairing concordantly. As a pair of protein-coding cis-sense/antisense transcripts, WDR83 and DHPS are upregulated simultaneously and correlate positively in lung cancer. They drive the pathophysiology of lung cancer by promoting cell proliferation (Su et al., 2012). Furthermore, the remaining SF PPIL1, which has not been directly reported in the literature to be associated with cancer, is a member of the peptidyl-prolyl isomerase procyclin family and is frequently overexpressed in colon cancer cells (Chai et al., 2021). In summary, it is reasonable to speculate that the 7 SFs may play a role in the development of tumors, and the relationship between these SFs and lung cancer warrants further exploration in the future.

Our analysis method can be used not only to screen for key SFs in LUSC but also to apply to a wider range of studies. From the perspective of the study object, although our method is only applied to LUSC data in this study, it is also applicable to other tumor data. From the perspective of research objectives, our method is not limited to screening SFs, but also can be used to screen regulatory factors, such as transcription factors, miRNAs or lncRNAs. For example, we can screen combinations of genes that can accurately classify tumor samples by downscaling or regression and then find key vertices by constructing a regulatory network of miRNAs that can anchor key miRNAs associated with tumors.

In conclusion, our analytical approach with a wide range of applications helps to obtain proper results and can provide new directions and perspectives for the exploration of related studies. In our study, although the specific functions and mechanisms of the 10 key SFs need to be further investigated, the available data and literature imply that they play a critical role in LUSC. Seven of these new SFs are also expected to be a new focus for future studies on SFs in LUSC. Furthermore, our proposed method will provide ideas and references for more studies. However, some limitations remain in our study. Due to the complexity of calculating multivariate combinations, we only calculated bivariate combinations, but multifactor combinations were not further explored. In subsequent studies, we will further improve our methods and extend to more scientific questions to provide novel focuses for future research.

## Conclusion

Abnormal AS is widely considered a novel indicator of carcinogenic processes, and SFs play a vital role in this process. Consequently, our aim is to screen key SFs that regulate carcinogenesis and progression. All combinations consisting of two ASEs were first constructed and screened using bivariate Cox regression and AUROC. Next, an association network of OS-ASEs and SFs was constructed by the Spearman correlation. Based on topological properties, we screened the top 10 SFs in terms of degree centrality. Finally, literature and data validation were performed on these 10 SFs. The data validation showed that 10 SFs were all significantly differentially expressed in both cancerous and paracancerous tissues of LUSC patients. Moreover, 5 of these SFs showed prognostic effects. It has been reported that 8 of these SFs are closely associated with tumors. In addition, cross-platform validations of GEO were carried out, and similar results were obtained. These findings can serve as a reference for subsequent experimental studies.

## Data Availability

Publicly available datasets were analyzed in this study. This data can be found here: TCGA database (https://portal.gdc.cancer.gov/projects/TCGA-LUSC), TCGASpliceSeq database (https://bioinformatics.mdanderson.org/TCGASpliceSeq/index.jsp).
