# Supplementary material for: Screening and Identification of Survival-Associated Splicing Factors in Lung Squamous Cell Carcinoma
Source: Front Genet. 2022 Jan 20;12:803606. doi: 10.3389/fgene.2021.803606 (PMC8811261; doi:10.3389/fgene.2021.803606)
Supplement: Supplementary file 1 [file Table1.DOCX]

Supplementary Material

# Supplementary Figures and Tables

## Supplementary Tables

### Supplementary Table 1：The information of GEO datasets

| **Datasets** | **Disease type** | **Experimental platform** | **Number of cases (tumor/normal)** |
| --- | --- | --- | --- |
| GSE157010 | LUSC | GPL570 | 235(235/0) |
| GSE3268 | LUSC | GPL96 | 10(5/5) |
| GSE6044 | LUSC | GPL201 | 20(5/15) |

### Supplementary Table 2：Information on 404 splicing factors

| **Number** | **SYMBOL** | **ENTREZID** | **ENSEMBL** |
| --- | --- | --- | --- |
| 1 | ACIN1 | 22985 | ENSG00000100813 |
| 2 | AGGF1 | 55109 | ENSG00000164252 |
| 3 | ALYREF | 10189 | ENSG00000183684 |
| 4 | AQR | 9716 | ENSG00000021776 |
| 5 | ARGLU1 | 55082 | ENSG00000134884 |
| 6 | BAG2 | 9532 | ENSG00000112208 |
| 7 | BCAS1 | 8537 | ENSG00000064787 |
| 8 | BCAS2 | 10286 | ENSG00000116752 |
| 9 | BUB3 | 9184 | ENSG00000154473 |
| 10 | BUD13 | 84811 | ENSG00000137656 |
| 11 | BUD31 | 8896 | ENSG00000106245 |
| 12 | NCBP3 | 55421 | ENSG00000074356 |
| 13 | TRIR | 79002 | ENSG00000123144 |
| 14 | SDE2 | 163859 | ENSG00000143751 |
| 15 | C1QBP | 708 | ENSG00000108561 |
| 16 | C9orf78 | 51759 | ENSG00000136819 |
| 17 | CACTIN | 58509 | ENSG00000105298 |
| 18 | CCAR1 | 55749 | ENSG00000060339 |
| 19 | CCDC12 | 151903 | ENSG00000160799 |
| 20 | CCDC130 | 81576 | ENSG00000104957 |
| 21 | GPATCH11 | 253635 | ENSG00000152133 |
| 22 | YJU2 | 55702 | ENSG00000105248 |
| 23 | CD2BP2 | 10421 | ENSG00000169217 |
| 24 | CDC40 | 51362 | ENSG00000168438 |
| 25 | CDC5L | 988 | ENSG00000096401 |
| 26 | CDK10 | 8558 | ENSG00000185324 |
| 27 | CDK11A | 728642 | ENSG00000008128 |
| 28 | CDK12 | 51755 | ENSG00000167258 |
| 29 | CELF1 | 10658 | ENSG00000149187 |
| 30 | CELF2 | 10659 | ENSG00000048740 |
| 31 | CELF3 | 11189 | ENSG00000159409 |
| 32 | CELF4 | 56853 | ENSG00000101489 |
| 33 | CELF5 | 60680 | ENSG00000161082 |
| 34 | CELF6 | 60677 | ENSG00000140488 |
| 35 | CFAP20 | 29105 | ENSG00000070761 |
| 36 | CHERP | 10523 | ENSG00000085872 |
| 37 | CIRBP | 1153 | ENSG00000099622 |
| 38 | CLASRP | 11129 | ENSG00000104859 |
| 39 | CLK1 | 1195 | ENSG00000013441 |
| 40 | CLK2 | 1196 | ENSG00000176444 |
| 41 | CLK3 | 1198 | ENSG00000179335 |
| 42 | CLK4 | 57396 | ENSG00000113240 |
| 43 | CLNS1A | 1207 | ENSG00000074201 |
| 44 | CPSF6 | 11052 | ENSG00000111605 |
| 45 | CRNKL1 | 51340 | ENSG00000101343 |
| 46 | CSN3 | 1448 | ENSG00000171209 |
| 47 | CTNNBL1 | 56259 | ENSG00000132792 |
| 48 | CWC15 | 51503 | ENSG00000150316 |
| 49 | CWC22 | 57703 | ENSG00000163510 |
| 50 | CWC25 | 54883 | ENSG00000273559 |
| 51 | CWC27 | 10283 | ENSG00000153015 |
| 52 | CXorf56 | 63932 | ENSG00000018610 |
| 53 | DDX1 | 1653 | ENSG00000079785 |
| 54 | DDX17 | 10521 | ENSG00000100201 |
| 55 | DDX18 | 8886 | ENSG00000088205 |
| 56 | DDX19A | 55308 | ENSG00000168872 |
| 57 | DDX19B | 11269 | ENSG00000157349 |
| 58 | DDX20 | 11218 | ENSG00000064703 |
| 59 | DDX21 | 9188 | ENSG00000165732 |
| 60 | DDX23 | 9416 | ENSG00000174243 |
| 61 | INTS6L | 203522 | ENSG00000165359 |
| 62 | DDX27 | 55661 | ENSG00000124228 |
| 63 | DDX39A | 10212 | ENSG00000123136 |
| 64 | DDX39B | 7919 | ENSG00000198563 |
| 65 | DDX3X | 1654 | ENSG00000215301 |
| 66 | DDX3Y | 8653 | ENSG00000067048 |
| 67 | DDX41 | 51428 | ENSG00000183258 |
| 68 | DDX42 | 11325 | ENSG00000198231 |
| 69 | DDX46 | 9879 | ENSG00000145833 |
| 70 | DDX5 | 1655 | ENSG00000108654 |
| 71 | DDX50 | 79009 | ENSG00000107625 |
| 72 | DDX6 | 1656 | ENSG00000110367 |
| 73 | ESS2 | 8220 | ENSG00000100056 |
| 74 | DHX15 | 1665 | ENSG00000109606 |
| 75 | DHX16 | 8449 | ENSG00000204560 |
| 76 | DHX30 | 22907 | ENSG00000132153 |
| 77 | DHX34 | 9704 | ENSG00000134815 |
| 78 | DHX35 | 60625 | ENSG00000101452 |
| 79 | DHX36 | 170506 | ENSG00000174953 |
| 80 | DHX38 | 9785 | ENSG00000140829 |
| 81 | DHX40 | 79665 | ENSG00000108406 |
| 82 | DHX57 | 90957 | ENSG00000163214 |
| 83 | DHX8 | 1659 | ENSG00000067596 |
| 84 | DHX9 | 1660 | ENSG00000135829 |
| 85 | DNAJC6 | 9829 | ENSG00000116675 |
| 86 | DNAJC8 | 22826 | ENSG00000126698 |
| 87 | EEF1A1 | 1915 | ENSG00000156508 |
| 88 | EFTUD2 | 9343 | ENSG00000108883 |
| 89 | EIF2S2 | 8894 | ENSG00000125977 |
| 90 | EIF3A | 8661 | ENSG00000107581 |
| 91 | EIF4A3 | 9775 | ENSG00000141543 |
| 92 | ELAVL1 | 1994 | ENSG00000066044 |
| 93 | ELAVL2 | 1993 | ENSG00000107105 |
| 94 | ELAVL3 | 1995 | ENSG00000196361 |
| 95 | ELAVL4 | 1996 | ENSG00000162374 |
| 96 | FAM32A | 26017 | ENSG00000105058 |
| 97 | FAM50A | 9130 | ENSG00000071859 |
| 98 | FAM50B | 26240 | ENSG00000145945 |
| 99 | CCNQ | 92002 | ENSG00000262919 |
| 100 | FMR1 | 2332 | ENSG00000102081 |
| 101 | FRA10AC1 | 118924 | ENSG00000148690 |
| 102 | FRG1 | 2483 | ENSG00000109536 |
| 103 | FUBP1 | 8880 | ENSG00000162613 |
| 104 | FUBP3 | 8939 | ENSG00000107164 |
| 105 | FUS | 2521 | ENSG00000089280 |
| 106 | GEMIN2 | 8487 | ENSG00000092208 |
| 107 | GEMIN5 | 25929 | ENSG00000082516 |
| 108 | RACK1 | 10399 | ENSG00000204628 |
| 109 | GPATCH1 | 55094 | ENSG00000076650 |
| 110 | GPATCH3 | 63906 | ENSG00000198746 |
| 111 | GPATCH8 | 23131 | ENSG00000186566 |
| 112 | GPKOW | 27238 | ENSG00000068394 |
| 113 | GRSF1 | 2926 | ENSG00000132463 |
| 114 | HNRNPA0 | 10949 | ENSG00000177733 |
| 115 | HNRNPA1 | 3178 | ENSG00000135486 |
| 116 | HNRNPA2B1 | 3181 | ENSG00000122566 |
| 117 | HNRNPA3 | 220988 | ENSG00000170144 |
| 118 | HNRNPAB | 3182 | ENSG00000197451 |
| 119 | HNRNPC | 3183 | ENSG00000092199 |
| 120 | HNRNPCL1 | 343069 | ENSG00000179172 |
| 121 | HNRNPD | 3184 | ENSG00000138668 |
| 122 | HNRNPDL | 9987 | ENSG00000152795 |
| 123 | HNRNPF | 3185 | ENSG00000169813 |
| 124 | HNRNPH1 | 3187 | ENSG00000169045 |
| 125 | HNRNPH2 | 3188 | ENSG00000126945 |
| 126 | HNRNPH3 | 3189 | ENSG00000096746 |
| 127 | HNRNPK | 3190 | ENSG00000165119 |
| 128 | HNRNPL | 3191 | ENSG00000104824 |
| 129 | HNRNPLL | 92906 | ENSG00000143889 |
| 130 | HNRNPM | 4670 | ENSG00000099783 |
| 131 | HNRNPR | 10236 | ENSG00000125944 |
| 132 | HNRNPU | 3192 | ENSG00000153187 |
| 133 | HNRNPUL1 | 11100 | ENSG00000105323 |
| 134 | HNRNPUL2 | 221092 | ENSG00000214753 |
| 135 | HSPA1A | 3303 | ENSG00000204389 |
| 136 | HSPA1B | 3304 | ENSG00000204388 |
| 137 | HSPA5 | 3309 | ENSG00000044574 |
| 138 | HSPA8 | 3312 | ENSG00000109971 |
| 139 | HSPB1 | 3315 | ENSG00000106211 |
| 140 | HTATSF1 | 27336 | ENSG00000102241 |
| 141 | IGF2BP3 | 10643 | ENSG00000136231 |
| 142 | IK | 3550 | ENSG00000113141 |
| 143 | ILF2 | 3608 | ENSG00000143621 |
| 144 | ILF3 | 3609 | ENSG00000129351 |
| 145 | INTS1 | 26173 | ENSG00000164880 |
| 146 | INTS3 | 65123 | ENSG00000143624 |
| 147 | INTS4 | 92105 | ENSG00000149262 |
| 148 | INTS5 | 80789 | ENSG00000185085 |
| 149 | INTS6 | 26512 | ENSG00000102786 |
| 150 | INTS7 | 25896 | ENSG00000143493 |
| 151 | ISY1 | 57461 | ENSG00000240682 |
| 152 | JUP | 3728 | ENSG00000173801 |
| 153 | KHDRBS1 | 10657 | ENSG00000121774 |
| 154 | KHDRBS3 | 10656 | ENSG00000131773 |
| 155 | KHSRP | 8570 | ENSG00000088247 |
| 156 | VIRMA | 25962 | ENSG00000164944 |
| 157 | CCAR2 | 57805 | ENSG00000158941 |
| 158 | KIN | 22944 | ENSG00000151657 |
| 159 | LENG1 | 79165 | ENSG00000105617 |
| 160 | HNRNPCL3 | 649330 | ENSG00000277058 |
| 161 | LSM1 | 27257 | ENSG00000175324 |
| 162 | LSM10 | 84967 | ENSG00000181817 |
| 163 | LSM2 | 57819 | ENSG00000204392 |
| 164 | LSM3 | 27258 | ENSG00000170860 |
| 165 | LSM4 | 25804 | ENSG00000130520 |
| 166 | LSM5 | 23658 | ENSG00000106355 |
| 167 | LSM6 | 11157 | ENSG00000164167 |
| 168 | LSM7 | 51690 | ENSG00000130332 |
| 169 | LSM8 | 51691 | ENSG00000128534 |
| 170 | NAA38 | 84316 | ENSG00000183011 |
| 171 | LUC7L | 55692 | ENSG00000007392 |
| 172 | LUC7L2 | 51631 | ENSG00000146963 |
| 173 | LUC7L3 | 51747 | ENSG00000108848 |
| 174 | MAGOH | 4116 | ENSG00000162385 |
| 175 | MATR3 | 9782 | ENSG00000015479 |
| 176 | MBNL1 | 4154 | ENSG00000152601 |
| 177 | MBNL2 | 10150 | ENSG00000139793 |
| 178 | MBNL3 | 55796 | ENSG00000076770 |
| 179 | MFAP1 | 4236 | ENSG00000140259 |
| 180 | MFSD11 | 79157 | ENSG00000092931 |
| 181 | MOV10 | 4343 | ENSG00000155363 |
| 182 | MSI1 | 4440 | ENSG00000135097 |
| 183 | MSI2 | 124540 | ENSG00000153944 |
| 184 | MYEF2 | 50804 | ENSG00000104177 |
| 185 | NCBP1 | 4686 | ENSG00000136937 |
| 186 | NCBP2 | 22916 | ENSG00000114503 |
| 187 | NELFE | 7936 | ENSG00000204356 |
| 188 | NKAP | 79576 | ENSG00000101882 |
| 189 | NONO | 4841 | ENSG00000147140 |
| 190 | NOSIP | 51070 | ENSG00000142546 |
| 191 | NOVA1 | 4857 | ENSG00000139910 |
| 192 | NOVA2 | 4858 | ENSG00000104967 |
| 193 | NRIP2 | 83714 | ENSG00000053702 |
| 194 | NSRP1 | 84081 | ENSG00000126653 |
| 195 | NUDT21 | 11051 | ENSG00000167005 |
| 196 | NUMA1 | 4926 | ENSG00000137497 |
| 197 | PABPC1 | 26986 | ENSG00000070756 |
| 198 | PAXBP1 | 94104 | ENSG00000159086 |
| 199 | PCBP1 | 5093 | ENSG00000169564 |
| 200 | PCBP2 | 5094 | ENSG00000197111 |
| 201 | PCBP3 | 54039 | ENSG00000183570 |
| 202 | PCBP4 | 57060 | ENSG00000090097 |
| 203 | PDCD7 | 10081 | ENSG00000090470 |
| 204 | PHF5A | 84844 | ENSG00000100410 |
| 205 | PLRG1 | 5356 | ENSG00000171566 |
| 206 | PNN | 5411 | ENSG00000100941 |
| 207 | PPIE | 10450 | ENSG00000084072 |
| 208 | PPIG | 9360 | ENSG00000138398 |
| 209 | PPIH | 10465 | ENSG00000171960 |
| 210 | PPIL1 | 51645 | ENSG00000137168 |
| 211 | PPIL2 | 23759 | ENSG00000100023 |
| 212 | PPIL3 | 53938 | ENSG00000240344 |
| 213 | PPIL4 | 85313 | ENSG00000131013 |
| 214 | PPM1G | 5496 | ENSG00000115241 |
| 215 | PPP1CA | 5499 | ENSG00000172531 |
| 216 | PPP1R8 | 5511 | ENSG00000117751 |
| 217 | PPWD1 | 23398 | ENSG00000113593 |
| 218 | PQBP1 | 10084 | ENSG00000102103 |
| 219 | PRCC | 5546 | ENSG00000143294 |
| 220 | PRMT5 | 10419 | ENSG00000100462 |
| 221 | PRPF18 | 8559 | ENSG00000165630 |
| 222 | PRPF19 | 27339 | ENSG00000110107 |
| 223 | PRPF3 | 9129 | ENSG00000117360 |
| 224 | PRPF31 | 26121 | ENSG00000105618 |
| 225 | PRPF38A | 84950 | ENSG00000134748 |
| 226 | PRPF38B | 55119 | ENSG00000134186 |
| 227 | PRPF39 | 55015 | ENSG00000185246 |
| 228 | PRPF4 | 9128 | ENSG00000136875 |
| 229 | PRPF40A | 55660 | ENSG00000196504 |
| 230 | PRPF40B | 25766 | ENSG00000110844 |
| 231 | PRPF4B | 8899 | ENSG00000112739 |
| 232 | PRPF6 | 24148 | ENSG00000101161 |
| 233 | PRPF8 | 10594 | ENSG00000174231 |
| 234 | PSEN1 | 5663 | ENSG00000080815 |
| 235 | PSIP1 | 11168 | ENSG00000164985 |
| 236 | PTBP1 | 5725 | ENSG00000011304 |
| 237 | PTBP2 | 58155 | ENSG00000117569 |
| 238 | PTBP3 | 9991 | ENSG00000119314 |
| 239 | PUF60 | 22827 | ENSG00000179950 |
| 240 | QKI | 9444 | ENSG00000112531 |
| 241 | RALY | 22913 | ENSG00000125970 |
| 242 | RALYL | 138046 | ENSG00000184672 |
| 243 | RAVER1 | 125950 | ENSG00000161847 |
| 244 | RAVER2 | 55225 | ENSG00000162437 |
| 245 | RBBP6 | 5930 | ENSG00000122257 |
| 246 | RBFOX2 | 23543 | ENSG00000100320 |
| 247 | RBM10 | 8241 | ENSG00000182872 |
| 248 | RBM14 | 10432 | ENSG00000239306 |
| 249 | RBM15 | 64783 | ENSG00000162775 |
| 250 | RBM15B | 29890 | ENSG00000259956 |
| 251 | RBM17 | 84991 | ENSG00000134453 |
| 252 | RBM22 | 55696 | ENSG00000086589 |
| 253 | RBM23 | 55147 | ENSG00000100461 |
| 254 | RBM25 | 58517 | ENSG00000119707 |
| 255 | RBM26 | 64062 | ENSG00000139746 |
| 256 | RBM27 | 54439 | ENSG00000091009 |
| 257 | RBM3 | 5935 | ENSG00000102317 |
| 258 | RBM39 | 9584 | ENSG00000131051 |
| 259 | RBM4 | 5936 | ENSG00000173933 |
| 260 | RBM42 | 79171 | ENSG00000126254 |
| 261 | RBM45 | 129831 | ENSG00000155636 |
| 262 | RBM47 | 54502 | ENSG00000163694 |
| 263 | RBM4B | 83759 | ENSG00000173914 |
| 264 | RBM5 | 10181 | ENSG00000003756 |
| 265 | RBM7 | 10179 | ENSG00000076053 |
| 266 | RBM8A | 9939 | ENSG00000265241 |
| 267 | RBMS1 | 5937 | ENSG00000153250 |
| 268 | RBMX | 27316 | ENSG00000147274 |
| 269 | RBMX2 | 51634 | ENSG00000134597 |
| 270 | RBMXL1 | 494115 | ENSG00000213516 |
| 271 | RBMXL2 | 27288 | ENSG00000170748 |
| 272 | RNF113A | 7737 | ENSG00000125352 |
| 273 | RNF20 | 56254 | ENSG00000155827 |
| 274 | RNF213 | 57674 | ENSG00000173821 |
| 275 | RNF34 | 80196 | ENSG00000170633 |
| 276 | RNF40 | 9810 | ENSG00000103549 |
| 277 | RNPC3 | 55599 | ENSG00000185946 |
| 278 | RNPS1 | 10921 | ENSG00000205937 |
| 279 | RNU1-1 | 26871 | ENSG00000206652 |
| 280 | RNU2-1 | 6066 | ENSG00000274585 |
| 281 | RNU4-1 | 26835 | ENSG00000200795 |
| 282 | RNU5A-1 | 26831 | ENSG00000199568 |
| 283 | RNU6-1 | 26827 | ENSG00000206625 |
| 284 | SAP18 | 10284 | ENSG00000150459 |
| 285 | SAP30BP | 29115 | ENSG00000161526 |
| 286 | SART1 | 9092 | ENSG00000175467 |
| 287 | SEC31B | 25956 | ENSG00000075826 |
| 288 | SF1 | 7536 | ENSG00000168066 |
| 289 | SF3A1 | 10291 | ENSG00000099995 |
| 290 | SF3A2 | 8175 | ENSG00000104897 |
| 291 | SF3A3 | 10946 | ENSG00000183431 |
| 292 | SF3B1 | 23451 | ENSG00000115524 |
| 293 | SF3B2 | 10992 | ENSG00000087365 |
| 294 | SF3B3 | 23450 | ENSG00000189091 |
| 295 | SF3B4 | 10262 | ENSG00000143368 |
| 296 | SF3B5 | 83443 | ENSG00000169976 |
| 297 | SF3B6 | 51639 | ENSG00000115128 |
| 298 | SFPQ | 6421 | ENSG00000116560 |
| 299 | MTREX | 23517 | ENSG00000039123 |
| 300 | SLU7 | 10569 | ENSG00000164609 |
| 301 | SMN1 | 6606 | ENSG00000172062 |
| 302 | SMNDC1 | 10285 | ENSG00000119953 |
| 303 | SMU1 | 55234 | ENSG00000122692 |
| 304 | SNIP1 | 79753 | ENSG00000163877 |
| 305 | SNRNP200 | 23020 | ENSG00000144028 |
| 306 | SNRNP25 | 79622 | ENSG00000161981 |
| 307 | SNRNP27 | 11017 | ENSG00000124380 |
| 308 | SNRNP35 | 11066 | ENSG00000184209 |
| 309 | SNRNP40 | 9410 | ENSG00000060688 |
| 310 | SNRNP48 | 154007 | ENSG00000168566 |
| 311 | SNRNP70 | 6625 | ENSG00000104852 |
| 312 | SNRPA | 6626 | ENSG00000077312 |
| 313 | SNRPA1 | 6627 | ENSG00000131876 |
| 314 | SNRPB | 6628 | ENSG00000125835 |
| 315 | SNRPB2 | 6629 | ENSG00000125870 |
| 316 | SNRPC | 6631 | ENSG00000124562 |
| 317 | SNRPD1 | 6632 | ENSG00000167088 |
| 318 | SNRPD2 | 6633 | ENSG00000125743 |
| 319 | SNRPD3 | 6634 | ENSG00000100028 |
| 320 | SNRPE | 6635 | ENSG00000182004 |
| 321 | SNRPF | 6636 | ENSG00000139343 |
| 322 | SNRPG | 6637 | ENSG00000143977 |
| 323 | SNRPN | 6638 | ENSG00000128739 |
| 324 | SNU13 | 4809 | ENSG00000100138 |
| 325 | SNURF | 8926 | ENSG00000273173 |
| 326 | SNW1 | 22938 | ENSG00000100603 |
| 327 | SPEN | 23013 | ENSG00000065526 |
| 328 | SREK1 | 140890 | ENSG00000153914 |
| 329 | SRPK1 | 6732 | ENSG00000096063 |
| 330 | SRPK2 | 6733 | ENSG00000135250 |
| 331 | SRPK3 | 26576 | ENSG00000184343 |
| 332 | SRRM1 | 10250 | ENSG00000133226 |
| 333 | SRRM2 | 23524 | ENSG00000167978 |
| 334 | SRRT | 51593 | ENSG00000087087 |
| 335 | SRSF1 | 6426 | ENSG00000136450 |
| 336 | SRSF10 | 10772 | ENSG00000188529 |
| 337 | SRSF11 | 9295 | ENSG00000116754 |
| 338 | SRSF12 | 135295 | ENSG00000154548 |
| 339 | SRSF2 | 6427 | ENSG00000161547 |
| 340 | SRSF3 | 6428 | ENSG00000112081 |
| 341 | SRSF4 | 6429 | ENSG00000116350 |
| 342 | SRSF5 | 6430 | ENSG00000100650 |
| 343 | SRSF6 | 6431 | ENSG00000124193 |
| 344 | SRSF7 | 6432 | ENSG00000115875 |
| 345 | SRSF8 | 10929 | ENSG00000263465 |
| 346 | SRSF9 | 8683 | ENSG00000111786 |
| 347 | SSB | 6741 | ENSG00000138385 |
| 348 | SUGP1 | 57794 | ENSG00000105705 |
| 349 | SYF2 | 25949 | ENSG00000117614 |
| 350 | SYNCRIP | 10492 | ENSG00000135316 |
| 351 | TAF15 | 8148 | ENSG00000270647 |
| 352 | TCERG1 | 10915 | ENSG00000113649 |
| 353 | TFIP11 | 24144 | ENSG00000100109 |
| 354 | THOC1 | 9984 | ENSG00000079134 |
| 355 | THOC2 | 57187 | ENSG00000125676 |
| 356 | THOC3 | 84321 | ENSG00000051596 |
| 357 | THOC5 | 8563 | ENSG00000100296 |
| 358 | THOC6 | 79228 | ENSG00000131652 |
| 359 | THOC7 | 80145 | ENSG00000163634 |
| 360 | THRAP3 | 9967 | ENSG00000054118 |
| 361 | TIA1 | 7072 | ENSG00000116001 |
| 362 | TIAL1 | 7073 | ENSG00000151923 |
| 363 | TNPO1 | 3842 | ENSG00000083312 |
| 364 | TOE1 | 114034 | ENSG00000132773 |
| 365 | TOP1MT | 116447 | ENSG00000184428 |
| 366 | TOPORS | 10210 | ENSG00000197579 |
| 367 | TRA2A | 29896 | ENSG00000164548 |
| 368 | TRA2B | 6434 | ENSG00000136527 |
| 369 | TRIM24 | 8805 | ENSG00000122779 |
| 370 | TTC14 | 151613 | ENSG00000163728 |
| 371 | TXNL4A | 10907 | ENSG00000141759 |
| 372 | U2AF1 | 7307 | ENSG00000160201 |
| 373 | U2AF1L4 | 199746 | ENSG00000161265 |
| 374 | U2AF2 | 11338 | ENSG00000063244 |
| 375 | U2SURP | 23350 | ENSG00000163714 |
| 376 | UBL5 | 59286 | ENSG00000198258 |
| 377 | USP39 | 10713 | ENSG00000168883 |
| 378 | WBP11 | 51729 | ENSG00000084463 |
| 379 | WBP4 | 11193 | ENSG00000120688 |
| 380 | WDR77 | 79084 | ENSG00000116455 |
| 381 | WDR83 | 84292 | ENSG00000123154 |
| 382 | WTAP | 9589 | ENSG00000146457 |
| 383 | XAB2 | 56949 | ENSG00000076924 |
| 384 | YBX1 | 4904 | ENSG00000065978 |
| 385 | YBX3 | 8531 | ENSG00000060138 |
| 386 | ZC3H11A | 9877 | ENSG00000058673 |
| 387 | ZC3H13 | 23091 | ENSG00000123200 |
| 388 | ZC3H18 | 124245 | ENSG00000158545 |
| 389 | ZC3H4 | 23211 | ENSG00000130749 |
| 390 | ZC3HAV1 | 56829 | ENSG00000105939 |
| 391 | ZCCHC10 | 54819 | ENSG00000155329 |
| 392 | ZCCHC8 | 55596 | ENSG00000033030 |
| 393 | ZCRB1 | 85437 | ENSG00000139168 |
| 394 | ZFR | 51663 | ENSG00000056097 |
| 395 | ZMAT2 | 153527 | ENSG00000146007 |
| 396 | ZMAT5 | 55954 | ENSG00000100319 |
| 397 | ZMYM3 | 9203 | ENSG00000147130 |
| 398 | ZNF131 | 7690 | ENSG00000172262 |
| 399 | ZNF207 | 7756 | ENSG00000010244 |
| 400 | ZNF326 | 284695 | ENSG00000162664 |
| 401 | ZNF346 | 23567 | ENSG00000113761 |
| 402 | ZNF830 | 91603 | ENSG00000198783 |
| 403 | ZRSR2P1 | 7310 | ENSG00000212643 |
| 404 | ZRSR2 | 8233 | ENSG00000169249 |

### Supplementary Table 3：The accession codes of TCGA of the 493 LUSC specimens

| TCGA-18-3406 | TCGA-34-5239 | TCGA-56-8309 | TCGA-66-2781 | TCGA-85-8664 |
| --- | --- | --- | --- | --- |
| TCGA-18-3407 | TCGA-34-5240 | TCGA-56-8503 | TCGA-66-2782 | TCGA-85-8666 |
| TCGA-18-3408 | TCGA-34-5241 | TCGA-56-8504 | TCGA-66-2783 | TCGA-85-A4CL |
| TCGA-18-3409 | TCGA-34-5927 | TCGA-56-8622 | TCGA-66-2785 | TCGA-85-A4CN |
| TCGA-18-3410 | TCGA-34-5928 | TCGA-56-8623 | TCGA-66-2786 | TCGA-85-A4JB |
| TCGA-18-3411 | TCGA-34-5929 | TCGA-56-8624 | TCGA-66-2787 | TCGA-85-A4JC |
| TCGA-18-3412 | TCGA-34-7107 | TCGA-56-8625 | TCGA-66-2788 | TCGA-85-A4PA |
| TCGA-18-3414 | TCGA-34-8454 | TCGA-56-8626 | TCGA-66-2789 | TCGA-85-A4QQ |
| TCGA-18-3415 | TCGA-34-8455 | TCGA-56-8628 | TCGA-66-2790 | TCGA-85-A4QR |
| TCGA-18-3416 | TCGA-34-8456 | TCGA-56-8629 | TCGA-66-2791 | TCGA-85-A50M |
| TCGA-18-3417 | TCGA-34-A5IX | TCGA-56-A49D | TCGA-66-2792 | TCGA-85-A50Z |
| TCGA-18-3419 | TCGA-37-3783 | TCGA-56-A4BW | TCGA-66-2793 | TCGA-85-A510 |
| TCGA-18-3421 | TCGA-37-3789 | TCGA-56-A4BX | TCGA-66-2794 | TCGA-85-A511 |
| TCGA-18-4083 | TCGA-37-3792 | TCGA-56-A4BY | TCGA-66-2795 | TCGA-85-A512 |
| TCGA-18-4086 | TCGA-37-4129 | TCGA-56-A4ZJ | TCGA-66-2800 | TCGA-85-A513 |
| TCGA-18-4721 | TCGA-37-4130 | TCGA-56-A4ZK | TCGA-68-7755 | TCGA-85-A53L |
| TCGA-18-5592 | TCGA-37-4132 | TCGA-56-A5DR | TCGA-68-7756 | TCGA-85-A5B5 |
| TCGA-18-5595 | TCGA-37-4133 | TCGA-56-A5DS | TCGA-68-7757 | TCGA-90-6837 |
| TCGA-21-1070 | TCGA-37-4135 | TCGA-56-A62T | TCGA-68-8250 | TCGA-90-7766 |
| TCGA-21-1071 | TCGA-37-4141 | TCGA-58-8386 | TCGA-68-8251 | TCGA-90-7767 |
| TCGA-21-1072 | TCGA-37-5819 | TCGA-58-8387 | TCGA-68-A59I | TCGA-90-7769 |
| TCGA-21-1075 | TCGA-37-A5EL | TCGA-58-8388 | TCGA-68-A59J | TCGA-90-7964 |
| TCGA-21-1076 | TCGA-37-A5EM | TCGA-58-8390 | TCGA-70-6722 | TCGA-90-A4ED |
| TCGA-21-1077 | TCGA-37-A5EN | TCGA-58-8391 | TCGA-70-6723 | TCGA-90-A4EE |
| TCGA-21-1078 | TCGA-39-5011 | TCGA-58-8392 | TCGA-77-6842 | TCGA-90-A59Q |
| TCGA-21-1079 | TCGA-39-5016 | TCGA-58-8393 | TCGA-77-6843 | TCGA-92-7340 |
| TCGA-21-1080 | TCGA-39-5019 | TCGA-58-A46J | TCGA-77-6844 | TCGA-92-7341 |
| TCGA-21-1081 | TCGA-39-5021 | TCGA-58-A46K | TCGA-77-6845 | TCGA-92-8063 |
| TCGA-21-1082 | TCGA-39-5022 | TCGA-58-A46L | TCGA-77-7138 | TCGA-92-8064 |
| TCGA-21-1083 | TCGA-39-5024 | TCGA-58-A46M | TCGA-77-7139 | TCGA-92-8065 |
| TCGA-21-5782 | TCGA-39-5027 | TCGA-58-A46N | TCGA-77-7140 | TCGA-94-7033 |
| TCGA-21-5783 | TCGA-39-5028 | TCGA-60-2695 | TCGA-77-7141 | TCGA-94-7557 |
| TCGA-21-5784 | TCGA-39-5029 | TCGA-60-2696 | TCGA-77-7142 | TCGA-94-7943 |
| TCGA-21-5786 | TCGA-39-5030 | TCGA-60-2697 | TCGA-77-7335 | TCGA-94-8035 |
| TCGA-21-5787 | TCGA-39-5031 | TCGA-60-2698 | TCGA-77-7337 | TCGA-94-8490 |
| TCGA-21-A5DI | TCGA-39-5034 | TCGA-60-2703 | TCGA-77-7338 | TCGA-94-8491 |
| TCGA-22-0940 | TCGA-39-5035 | TCGA-60-2704 | TCGA-77-7463 | TCGA-94-A4VJ |
| TCGA-22-0944 | TCGA-39-5036 | TCGA-60-2706 | TCGA-77-7465 | TCGA-94-A5I4 |
| TCGA-22-1000 | TCGA-39-5037 | TCGA-60-2707 | TCGA-77-8007 | TCGA-94-A5I6 |
| TCGA-22-1002 | TCGA-39-5039 | TCGA-60-2708 | TCGA-77-8008 | TCGA-96-7544 |
| TCGA-22-1005 | TCGA-39-5040 | TCGA-60-2709 | TCGA-77-8009 | TCGA-96-7545 |
| TCGA-22-1011 | TCGA-43-2576 | TCGA-60-2710 | TCGA-77-8128 | TCGA-96-8169 |
| TCGA-22-1012 | TCGA-43-2578 | TCGA-60-2711 | TCGA-77-8130 | TCGA-96-8170 |
| TCGA-22-1016 | TCGA-43-2581 | TCGA-60-2712 | TCGA-77-8131 | TCGA-96-A4JK |
| TCGA-22-1017 | TCGA-43-3394 | TCGA-60-2713 | TCGA-77-8133 | TCGA-96-A4JL |
| TCGA-22-4591 | TCGA-43-3920 | TCGA-60-2714 | TCGA-77-8136 | TCGA-98-7454 |
| TCGA-22-4593 | TCGA-43-5668 | TCGA-60-2715 | TCGA-77-8138 | TCGA-98-8020 |
| TCGA-22-4594 | TCGA-43-5670 | TCGA-60-2716 | TCGA-77-8139 | TCGA-98-8021 |
| TCGA-22-4595 | TCGA-43-6143 | TCGA-60-2719 | TCGA-77-8140 | TCGA-98-8022 |
| TCGA-22-4596 | TCGA-43-6647 | TCGA-60-2720 | TCGA-77-8143 | TCGA-98-8023 |
| TCGA-22-4599 | TCGA-43-6770 | TCGA-60-2721 | TCGA-77-8144 | TCGA-98-A538 |
| TCGA-22-4601 | TCGA-43-6771 | TCGA-60-2722 | TCGA-77-8145 | TCGA-98-A539 |
| TCGA-22-4604 | TCGA-43-6773 | TCGA-60-2723 | TCGA-77-8146 | TCGA-98-A53A |
| TCGA-22-4605 | TCGA-43-7656 | TCGA-60-2724 | TCGA-77-8148 | TCGA-98-A53B |
| TCGA-22-4607 | TCGA-43-7657 | TCGA-60-2725 | TCGA-77-8150 | TCGA-98-A53C |
| TCGA-22-4609 | TCGA-43-7658 | TCGA-60-2726 | TCGA-77-8153 | TCGA-98-A53D |
| TCGA-22-4613 | TCGA-43-8115 | TCGA-63-6202 | TCGA-77-8154 | TCGA-98-A53H |
| TCGA-22-5471 | TCGA-43-8116 | TCGA-63-7020 | TCGA-77-8156 | TCGA-98-A53I |
| TCGA-22-5472 | TCGA-43-8118 | TCGA-63-7021 | TCGA-77-A5FZ | TCGA-98-A53J |
| TCGA-22-5473 | TCGA-43-A474 | TCGA-63-7022 | TCGA-77-A5G1 | TCGA-J1-A4AH |
| TCGA-22-5474 | TCGA-43-A475 | TCGA-63-7023 | TCGA-77-A5G3 | TCGA-L3-A4E7 |
| TCGA-22-5477 | TCGA-43-A56U | TCGA-63-A5MB | TCGA-77-A5G6 | TCGA-L3-A524 |
| TCGA-22-5478 | TCGA-43-A56V | TCGA-63-A5MG | TCGA-77-A5G7 | TCGA-LA-A446 |
| TCGA-22-5479 | TCGA-46-3765 | TCGA-63-A5MH | TCGA-77-A5G8 | TCGA-LA-A7SW |
| TCGA-22-5480 | TCGA-46-3766 | TCGA-63-A5MI | TCGA-77-A5GA | TCGA-MF-A522 |
| TCGA-22-5481 | TCGA-46-3767 | TCGA-63-A5MJ | TCGA-77-A5GF | TCGA-NC-A5HD |
| TCGA-22-5482 | TCGA-46-3768 | TCGA-63-A5ML | TCGA-77-A5GH | TCGA-NC-A5HE |
| TCGA-22-5483 | TCGA-46-3769 | TCGA-63-A5MM | TCGA-85-6175 | TCGA-NC-A5HF |
| TCGA-22-5485 | TCGA-46-6025 | TCGA-63-A5MN | TCGA-85-6560 | TCGA-NC-A5HG |
| TCGA-22-5489 | TCGA-46-6026 | TCGA-63-A5MP | TCGA-85-6561 | TCGA-NC-A5HH |
| TCGA-22-5491 | TCGA-51-4079 | TCGA-63-A5MR | TCGA-85-6798 | TCGA-NC-A5HI |
| TCGA-22-5492 | TCGA-51-4080 | TCGA-63-A5MS | TCGA-85-7696 | TCGA-NC-A5HJ |
| TCGA-22-A5C4 | TCGA-51-4081 | TCGA-63-A5MT | TCGA-85-7697 | TCGA-NC-A5HK |
| TCGA-33-4532 | TCGA-51-6867 | TCGA-63-A5MV | TCGA-85-7698 | TCGA-NC-A5HL |
| TCGA-33-4533 | TCGA-52-7622 | TCGA-63-A5MW | TCGA-85-7699 | TCGA-NC-A5HM |
| TCGA-33-4538 | TCGA-52-7809 | TCGA-63-A5MY | TCGA-85-7710 | TCGA-NC-A5HN |
| TCGA-33-4547 | TCGA-52-7810 | TCGA-66-2727 | TCGA-85-7843 | TCGA-NC-A5HO |
| TCGA-33-4566 | TCGA-52-7811 | TCGA-66-2734 | TCGA-85-7844 | TCGA-NC-A5HP |
| TCGA-33-4582 | TCGA-52-7812 | TCGA-66-2737 | TCGA-85-7950 | TCGA-NC-A5HQ |
| TCGA-33-4583 | TCGA-56-1622 | TCGA-66-2742 | TCGA-85-8048 | TCGA-NC-A5HR |
| TCGA-33-4586 | TCGA-56-5897 | TCGA-66-2744 | TCGA-85-8049 | TCGA-NC-A5HT |
| TCGA-33-4587 | TCGA-56-5898 | TCGA-66-2753 | TCGA-85-8052 | TCGA-NK-A5CR |
| TCGA-33-4589 | TCGA-56-6545 | TCGA-66-2754 | TCGA-85-8070 | TCGA-NK-A5CT |
| TCGA-33-6737 | TCGA-56-7221 | TCGA-66-2755 | TCGA-85-8071 | TCGA-NK-A5CX |
| TCGA-33-6738 | TCGA-56-7222 | TCGA-66-2756 | TCGA-85-8072 | TCGA-NK-A5D1 |
| TCGA-33-A4WN | TCGA-56-7223 | TCGA-66-2757 | TCGA-85-8276 | TCGA-NK-A7XE |
| TCGA-33-A5GW | TCGA-56-7579 | TCGA-66-2758 | TCGA-85-8277 | TCGA-O2-A52N |
| TCGA-33-AAS8 | TCGA-56-7580 | TCGA-66-2759 | TCGA-85-8287 | TCGA-O2-A52Q |
| TCGA-33-AASB | TCGA-56-7582 | TCGA-66-2763 | TCGA-85-8288 | TCGA-O2-A52S |
| TCGA-33-AASD | TCGA-56-7730 | TCGA-66-2765 | TCGA-85-8350 | TCGA-O2-A52V |
| TCGA-33-AASI | TCGA-56-7731 | TCGA-66-2766 | TCGA-85-8351 | TCGA-O2-A52W |
| TCGA-33-AASJ | TCGA-56-7822 | TCGA-66-2767 | TCGA-85-8352 | TCGA-O2-A5IB |
| TCGA-33-AASL | TCGA-56-7823 | TCGA-66-2768 | TCGA-85-8353 | TCGA-XC-AA0X |
| TCGA-34-2596 | TCGA-56-8082 | TCGA-66-2769 | TCGA-85-8354 |  |
| TCGA-34-2600 | TCGA-56-8083 | TCGA-66-2770 | TCGA-85-8355 |  |
| TCGA-34-2608 | TCGA-56-8201 | TCGA-66-2771 | TCGA-85-8479 |  |
| TCGA-34-5231 | TCGA-56-8304 | TCGA-66-2773 | TCGA-85-8481 |  |
| TCGA-34-5232 | TCGA-56-8305 | TCGA-66-2777 | TCGA-85-8580 |  |
| TCGA-34-5234 | TCGA-56-8307 | TCGA-66-2778 | TCGA-85-8582 |  |
| TCGA-34-5236 | TCGA-56-8308 | TCGA-66-2780 | TCGA-85-8584 |  |

### Supplementary Table 4：The correspondence between factor and ASE

（See TableS4.csv）

## Supplementary Figures

**Supplementary Figure 1.** The degree distribution of 398 splicing factors.

**Supplementary Figure 2.** KM curves demonstrating the ability of the SFs in the TCGA dataset to distinguish between high and low risk LUSC patients. The yellow curve represents the patients with high SF expression, and the blue curve represents the patients with low SF expression.

**Supplementary Figure 3.** KM curves demonstrating the ability of the SFs in the GSE157010 dataset to distinguish between high and low risk LUSC patients. The yellow curve represents the patients with high SF expression, and the blue curve represents the patients with low SF expression.

**Supplementary Figure 4.** The expression distribution of the matched 6 SFs in the GSE3268 dataset. The red color represents tumor samples and the grey color represents normal samples.

**Supplementary Figure 5.** The expression distribution of the matched 4 SFs in the GSE6044 dataset. The grey color represents normal samples, the dark blue color represents samples that did not receive platinum-based therapy and the light blue color represents samples that received.

### 1.3 Supplementary Source Code

### Supplementary Source Code1. Perl script for removing alternative splicing events with “null”

#!/usr/bin/perl

use strict;

use warnings;

use Getopt::Long;

use Pod::Usage;

my $input_file = 'LUSC.rawdata.clinical.01';

my $out_file = 'LUSC.rawdata.clinical.01.noNULL';

my $man = 0;

my $help = 0;

GetOptions(

'help|?' => \$help,

'man' => \$man,

'input=s' => \$input_file,

'output=s' => \$out_file,

) or pod2usage(2);

pod2usage(1) if $help;

pod2usage( -exitstatus => 0, -verbose => 2 ) if $man;

$|++;

open IN, "< $input_file";

open OUT, "> $out_file";

while ( my $data_line = <IN> ){

if( $data_line =~ /null/ ){ next; }

print OUT "$data_line";

}

close IN;

close OUT;

Supplementary Source Code2. Perl script for removing alternative splicing events with variance less than 0.001

#!/usr/bin/perl

use strict;

use warnings;

use Getopt::Long;

use Pod::Usage;

my $input_file = '2comb.cox';

my $out_file = '2comb.05.cox';

my $var_thred = 0.001;

my $man = 0;

my $help = 0;

GetOptions(

'help|?' => \$help,

'man' => \$man,

'input=s' => \$input_file,

'output=s' => \$out_file,

'var_thred=s' => \$var_thred

) or pod2usage(2);

pod2usage(1) if $help;

pod2usage( -exitstatus => 0, -verbose => 2 ) if $man;

$|++;

open IN, "< $input_file";

open OUT, "> $out_file";

foreach my $i (0..3){

my $data_line = <IN>;

print OUT "$data_line";

}

open TEMP, "> temp";

while ( my $data_line = <IN> ){

my @datas = split /\t/, $data_line;

my $id = shift @datas;

my $v = variance(@datas);

if ($v >= $var_thred ){

print OUT "$data_line";

}

print TEMP "$id\t$v\n";

}

close TEMP;

close IN;

close OUT;

sub average{

my $sum = 0;

my $count = scalar @_;

foreach (@_){

$sum += $_;

}

return $sum/$count;

}

sub variance{

my $mean = average(@_);

my $df = scalar @_ - 1;

my $ss = 0;

foreach (@_){

$ss += ($_ - $mean) * ($_ - $mean);

}

return $ss/$df;

}

**Supplementary Source Code3.** R script for KM analysis

library(ggplot2)

library(data.table)

library(survival)

library(survminer)

library(dplyr)

setwd("path")

exp <- fread("FILE",data.table = F)

mut <- exp[,which(colnames(exp) %in% c("time","event","gene"))]

res.cut <- surv_cutpoint(mut, time = "time", event = "event", variables = c("gene"))

res.cat <- surv_categorize(res.cut)

fit <- survfit(Surv(time, event) ~ gene, data = res.cat)

ggsurvplot(fit, main = "Survival curve"

, data = res.cat

, risk.table = F

, conf.int = F # confident internal

, pval = T

, pval.method = F

, legend.title = "gene"

, legend.labs=c("high","low")

, font.main = c(16, "bold", "darkblue")

, font.x = c(14, "bold", "black")

, font.y = c(14, "bold", "black")

, font.tickslab = c(12, "plain", "black")

,palette =c("#E7B800", "#2E9FDF")

, ggtheme = theme_bw()

)
